# Draft Genome Sequences of Two Strains of *Xanthomonas arboricola* pv. celebensis Isolated from Banana Plants

**DOI:** 10.1128/genomeA.01705-15

**Published:** 2016-02-11

**Authors:** James Harrison, Murray R. Grant, David J. Studholme

**Affiliations:** School of Biosciences, University of Exeter, Devon, United Kingdom

## Abstract

We report here the annotated draft genome sequences of strains *Xanthomonas arboricola* pv. celebensis NCPPB 1832 and NCPPB 1630 (NCPPB, National Collection of Plant Pathogenic Bacteria), both isolated from *Musa* species in New Zealand. This will allow the comparison of genomes between phylogenetically distant xanthomonads that have independently converged with the ability to colonize banana plants.

## GENOME ANNOUNCEMENT

The bacterial genus *Xanthomonas* contains pathogens and commensals that collectively infect hundreds of plant species (1). Within the genus, the ability to colonize banana (*Musa* species) has evolved at least three times. *Xanthomonas campestris* pv. musacearum is responsible for banana *Xanthomonas* wilt in Africa (2). *Xanthomonas arboricola* pv. celebensis has been found in India and Indonesia (including Sulawesi) and causes drooping and chlorotic and necrotic stripes on banana leaves; bacteria can spread through the vascular system and attack the rhizome (1). It may cause rotting of the rhizome and fruit. Milder chronic infections sometimes follow acute epidemics with severe attacks, resulting in death of the plant (3). This pathogen has been described as “*Xanthomonas musciola*” and as “*Xanthomonas celebensis*” (1, 3). Finally, *Xanthomonas* strains have been isolated from bananas in eastern and western Samoa that were not assigned to a named species. Genome sequences are already available for *X. campestris* pv. musacearum (4, 5) and for two Samoan *Xanthomonas* strains (6) but not for *X. arboricola* pv. celebensis. Genomic comparisons of these three phylogenetically disparate xanthomonads might yield insights into common strategies for colonizing bananas; therefore, we sequenced the genomes of two strains of *X. arboricola* pv. celebensis. Genome sequences (7–11) are available for several other strains and pathovars within the species *X. arboricola*, offering the possibility of identifying genomic features unique to the banana-pathogenic *X. arboricola* pv. celebensis.

Strains NCPPB 1630 and NCPPB 1832 were obtained from the National Collection of Plant Pathogenic Bacteria (NCPPB) at York in the United Kingdom. Both were originally isolated in 1960 by D. W. Dye from *Musa* species in New Zealand. The pathotype strain is NCPPB 1832 and is synonymous with LMG 677, ATCC 19045, PDDCC 1488, and ICPB XC145. We used the Illumina HiSeq to generate 9.7 million pairs of 100-bp reads for NCPPB 1630 and 2 million pairs of 100-bp reads for NCPPB 1832; raw data are available in the Sequence Read Archive (12). *De novo* assembly with Velvet version 1.2.10 (13) resulted in 3 scaffolds comprising a total of 75 contigs for NCPPB 1832. For the NCPPB 1630 assembly, there were 7 scaffolds, comprising a total of 121 contigs. The contig *N*_50_ lengths for NCPPB 1832 and NCPPB 1630 were 172,772 and 80,994 bp, respectively. Both genomes were annotated using the NCBI Prokaryotic Genome Annotation Pipeline (14) version 2.6 (rev. 439576).

### Nucleotide sequence accession numbers.

These whole-genome shotgun projects have been deposited in DDBJ/ENA/GenBank under the accession numbers JPHC00000000 (pathotype strain NCPPB 1832) and JPHE00000000 (strain NCPPB 1630). The versions described in this paper are the first versions, JPHC01000000 and JPHE01000000, respectively.
